# Temporal evolution of dermonecrosis in loxoscelism assessed by photodocumentation

**DOI:** 10.1590/0037-8682-0502-2021

**Published:** 2022-02-25

**Authors:** Carla Fernanda Borrasca-Fernandes, Camila Carbone Prado, Eduardo Mello De Capitani, Stephen Hyslop, Fábio Bucaretchi

**Affiliations:** 1 Universidade Estadual de Campinas, Faculdade de Ciências Médicas, Centro de Informação e Assistência Toxicológica de Campinas, Campinas, SP, Brasil.; 2 Universidade Estadual de Campinas, Faculdade de Ciências Médicas, Departamento de Clínica Médica, Campinas, SP, Brasil.; 3 Universidade Estadual de Campinas, Faculdade de Ciências Médicas, Departamento de Medicina Translacional (Área de Farmacologia), Campinas, SP, Brasil.; 4 Universidade Estadual de Campinas, Faculdade de Ciências Médicas, Departamento de Pediatria, Campinas, SP, Brasil.

**Keywords:** Antivenom, Brown spider, Dermonecrosis, *Loxosceles* spp., Loxoscelism

## Abstract

**Background:**

Although loxoscelism (bites by brown spiders of the genus *Loxosceles*) frequently results in dermonecrosis, no previous clinical reports have provided detailed temporal photodocumentation of the evolution of dermonecrotic lesions in a case series.

**Methods:**

This was a retrospective cohort study involving a case series of loxoscelism. Only cases of dermonecrosis with photodocumentation of lesion evolution (from admission until complete or almost complete healing) were included.

**Results:**

Eight patients (six men, two women; median age, 38 years) fulfilled the inclusion criteria. The bite sites included the thigh (n = 4), forearm (n = 2), abdomen (n = 1), and trunk (n = 1). Time interval between the bite and first contact with our service ranged from 15 to 216 h (median = 29 h). The main clinical manifestations included local erythematous and ischemic violaceous lesions overlying a base of indurated edema (livedoid plaque, 8), local pain (8), exanthema (6), serohemorrhagic vesicles/blisters (5), fever (5), and jaundice (1). Based on a previously established classification, the cases were classified as probable cutaneous-necrotic loxoscelism (CNL, n = 4), presumptive CNL (n = 3), and presumptive cutaneous-hemolytic loxoscelism (n = 1). Seven patients were treated with anti-arachnidic antivenom (AV; median time post-bite = 46 h). Complete lesion healing ranged from 34 to 98 days post-bite (median, 68 days; six patients). None of the patients required reconstructive plastic surgery.

**Conclusions:**

The sequential photographic documentation showed considerable variation in the process of wound healing, with complete epithelialization requiring up to 3 months after the bite.

## INTRODUCTION

Brown spiders of the genus *Loxosceles* (Heineken and Lowe, 1832) are globally distributed in temperate and tropical regions^1^ ^-^ ^3^. Bites by *Loxosceles* spp., clinically referred to as loxoscelism, are the main cause of envenomation by spiders of medical importance in Brazil and may result in local dermonecrosis with gravitational spreading (cutaneous-necrotic loxoscelism). On rare occasions, severe systemic complications, such as massive intravascular hemolysis and acute kidney injury (AKI) (cutaneous-hemolytic loxoscelism) may occur. Although at least 19 species of *Loxosceles* have been described in Brazil, most bites occur in southern and southeastern Brazil and are caused by *L. intermedia*, *L. laeta*, and *L. gaucho* ^2^ ^-^ ^4^. 

Loxoscelism diagnosis is difficult and is rarely based on the identification of the offending spider (<15% of cases in Brazil)^2^. Instead, diagnosis is based on epidemiological data and the known presence of *Loxosceles* spp. in the geographic region where the bite occurred, the patient´s clinical history, and the evolution of local/systemic manifestations^1^ ^-^ ^7^. Moreover, since at least 40 medical conditions, most of which are dermal infections, may be misdiagnosed as cutaneous-necrotic loxoscelism, Stoecker et al. (2017) developed a useful mnemonic device, referred to as ‘NOT RECLUSE’, that summarizes the typical and atypical findings of skin lesions caused by *L. reclusa* envenomation, the endemic *Loxosceles* species found in the south-central United States^8^.

In most cases, there is little or no pain associated with the initial envenomation, but algesia increases over time, with bitten individuals generally seeking medical attention 24-48 h post-bite when the cutaneous lesion starts to develop. Clinical indications of cutaneous loxoscelism include the presence of indurated edema under an irregularly demarcated region of ischemic pale skin, mingled with ecchymotic and hemorrhagic tissue that resembles local vasculitis, also known as a livedoid plaque, marble plaque or “white, red, blue/purple sign.” The presence of violaceous discoloration is considered an indication of incipient dermonecrosis^6^. The bite region may be associated with a local erythematous plaque, in addition to the presence of vesicles and blisters containing serous or hemorrhagic fluid. These lesions may evolve to dermonecrosis within a few days, with more severe lesions often observed in anatomical areas that have a higher concentration of fatty tissue, such as the thighs, buttocks, and abdomen^1^ ^-^ ^7^. 

The systemic manifestations associated with cutaneous loxoscelism include flu-like symptoms such as hyperthermia, malaise, headache, and myalgia, in addition to the appearance of local and generalized rashes, primarily as a scarlatiniform (the most common) or morbilliform rash. Patients with intravascular hemolysis may develop complications such as severe anemia, AKI, disseminated intravascular coagulation, rhabdomyolysis, and systemic shock, with some patients requiring a transfusion of red blood cell concentrate and renal replacement therapy^1^ ^-^ ^4^ ^,^ ^7^.

Despite the high frequency of loxoscelism in North and South America, to our knowledge, no previous clinical reports have provided detailed photodocumentation of the complete course of dermonecrosis (from admission until complete wound healing) in a case series of patients bitten by *Loxosceles* spp. In view of this limited documentation in clinical literature, in this report, we provide a comprehensive analysis of a case series of loxoscelism in which we use photodocumentation to describe the temporal clinical evolution of the dermal lesions and present the treatments used. These cases were observed and treated in the emergency department (ED) of the university teaching hospital at the State University of Campinas (UNICAMP), Campinas, São Paulo state, southeastern Brazil, and were followed up by the local poison control center (PCC) of the same institution.

## METHODS

### Patients and data collection

A retrospective cohort study was performed to evaluate a case series of patients with loxoscelism admitted to our ED from January 2008 to December 2019. Only cases that evolved to dermonecrosis with detailed photodocumentation of lesion evolution (from admission until complete or almost complete healing of the lesion) were included in the study; complete healing was defined based on the contiguity of the cutaneous surface with no exudate^6^. Clinical information for each case was retrieved from paper and electronic medical records and was compiled in a specific data system. All patients had their skin lesions photographed during the follow-up. Initial photodocumentation of the lesions was obtained from photographs sent to the PCC during the first call from a health service or patients/relatives, or from photographs taken upon case presentation at our ED. During follow-up, photographs were taken during in-person consultation or sent by the patients using a mobile phone app. All participants in the study provided formal informed consent for the use of the images in publication, and anonymity was guaranteed. This study was approved by the Institutional Committee for Ethics in Research (CEP-UNICAMP, protocol CAAE 97428818.5.0000.5404).

### Clinical classification

The cases were classified as loxoscelism based on the proposal by Sams et al.^5^ in one of the following categories: (1) documented or proven envenomation - offending spider found after bite identified by qualified person, typical lesion, and typical clinical course; (2) probable envenomation - spiders found in the area, patient may have felt the bite, the offending spider was seen, typical lesion, and typical clinical course; and (3) presumptive envenomation - spiders known to occur in the area, compatible lesion, and a typical clinical course. Based on the clinical presentation, patients who developed dermonecrosis only were classified as having cutaneous-necrotic loxoscelism (CNL), while those who developed dermonecrosis and clinical and laboratory features indicative of hemolysis were classified as having cutaneous-hemolytic loxoscelism (CHL)^2^ ^,^ ^3^. The spiders were identified to the genus level by co-author FB (physician) using a stereomicroscope to identify the U-shaped arrangement of the three pairs of eyes on the cephalothorax. Species-level identification was performed by an expert arachnologist in the Laboratory for Arthropods at the Instituto Butantan (São Paulo, SP, Brazil).

### Antivenom therapy

The use of anti-arachnidic antivenom [AV; soro antiaracnídico, Instituto Butantan, Brazil; equine origin; 5 mL/vial containing F(ab´)_2_ antibodies against *Loxosceles gaucho* (brown spider), *Phoneutria nigriventer* (wandering spider) and *Tityus serrulatus* (yellow scorpion) venoms; 1 mL of AV neutralizes 15 minimum necrotizing doses of reference *L. gaucho* venom in rabbits], was based on the Brazilian Ministry of Health guidelines^9^ ^,^ ^10^. The current guidelines recommend five vials of AV for patients who develop a severe local lesion, that is, a livedoid plaque with largest dimension >3 cm, including the surrounding indurated edema, and 10 vials for patients who develop hemolysis^10^. The AV was diluted in 0.9% saline and administered intravenously over 30-40 min, without prior medication. 

### Data analysis

For each case, we recorded the relevant information on individual files and on a spreadsheet (Excel, Microsoft Office 2010) prepared for the study; this included sociodemographic data, the spider genus or species (when identified), the local and systemic clinical manifestations of envenomation, time interval between the bite and AV administration, and the procedures used in the topical treatment of the lesions. Demographic and clinical data were tabulated as actual numbers for categorical variables, and as the median and 25^th^ and 75^th^ percentiles (interquartile range, IQR) for continuous variables.

## RESULTS

### Patient characteristics

During the study period, 76 patients diagnosed with loxoscelism were admitted to our ED and were monitored by PCC. Based on a detailed review of these cases by two of the authors (CFBF and FB), 68 patients were excluded from further analysis because 36 of them did not develop dermonecrosis and, of the 40 cases that did, 32 had incomplete photographic documentation of the lesion evolution. Thus, only eight cases fulfilled the criterion of complete photodocumentation from admission until complete or almost complete wound healing.

Table 1 summarizes the demographic data of the eight patients and the circumstances of the bites. Six cases involved men, with a median age of 38 years (IQR: 33-49 years), and the time between the bite and the first call to the PCC ranged from 15 to 216 h (median = 29 h, IQR: 22-60 h). Bites occurred on the thigh (4), forearm/arm (2), abdomen (1), and trunk (1). Three patients saw the offending brown spider at the time of the bite, with one of them bringing the offending animal for identification (case 8); however, even with the use of a stereomicroscope, spider identification was not possible because of extensive damage to the specimen. In case 2, a non-offending *Loxosceles* spp. was found inside the patient´s house, and in case 3, a non-offending brown spider found close to where the patient was bitten was identified as *L. gaucho* by the Laboratory for Arthropods at the Instituto Butantan (São Paulo, SP, Brazil).

**TABLE 1:** Demographic data and circumstances of the bites in eight cases of dermonecrotic loxoscelism.

Case	1	2	3	4	5	6	7	8	Median (IQR)
Age (years)	52	19	38	28	52	45	35	37	38 (33-49)
Sex	M	F	M	F	M	M	M	M	
Geographic setting	Rural	Urban	Rural	Urban	Rural	Rural	Urban	Urban	
Interval (h) between bite and first contact with the PCC	20	34	23	40	15	120	216	23	29 (22-60)
Bite site	Right pectoral	Left arm	Right thigh	Right thigh	Left flank	Right thigh	Left forearm	Left thigh	
Saw the offending spider	No	No	Yes	No	No	No	Yes	Yes	
Circumstances of the bite	Sleeping under a tree	Putting on a coat; brought a non-offending brown spider found inside her house	Doing carpentry; brought a non-offending brown spider found close to where he was bitten	Sleeping	Handling a bunch of bananas	Working on a farm	Gardening. Saw a brown spider upon removing his shirt	Hanging clothes on a washing line; brought the offending brown spider	
Spider identified	No	*Loxosceles* spp.	*L. gaucho*	No	No	No	No	No (spider too damaged to allow identification)	
Bite classification	Presumptive CNL	Probable CNL	Probable CNL	Presumptive CNL	Presumptive CHL	Presumptive CNL	Probable CNL	Probable CNL	

CHL: cutaneous hemolytic loxoscelism; CNL: cutaneous necrotic loxoscelism; F: female; IQR: interquartile range; M: male; PCC: poison control center.

### Clinical findings

Table 2 summarizes the clinical findings upon admission/evolution and subsequent treatments. Although the bites were initially accompanied by little or no local pain in most cases, in five cases there was a progressive development of pain that was intense by the time of admission, with all the skin lesions evolving to a livedoid plaque with a violaceous discoloration. The largest livedoid plaque dimension was >3 cm in six cases (median = 5 cm), excluding the surrounding indurated edema. Although the size of the lesion in the ischemic area was not assessed upon admission in cases 1 and 4, both developed a livedoid plaque >3 cm (Supplementary Material Figures 1,2,3 and 4). In addition, six patients showed erythema, six developed exanthema, and five developed vesicles/blisters. One patient (case 5) developed jaundice 3 days post-bite, with indirect hyperbilirubinemia (3.69 mg/dL; reference value for total bilirubin: 0.3-1.2 mg/dL) and a slight decrease in hemoglobin levels during evolution (admission = 13.3 g/dL; D3 = 11.1 g/dL). There were no changes in coagulation, complement components C3 and C4, and the reticulocyte count, or in the serum aminotransferase, creatinine, total CK, and lactate dehydrogenase activities or levels. Evidence of local skin ulceration was detected 7-26 days post-bite (median = 12 days). None of the patients developed secondary infections. Based on the above features, the eight cases were classified as probable CNL (n = 4), presumptive CNL (n = 3), and presumptive CHL (n = 1) (Tables 1 and 2).

**TABLE 2:** Clinical findings upon admission/evolution and the treatment given in eight cases of dermonecrotic loxoscelism.

Case	1	2	3	4	5	6	7	8	N	Median (IQR)
**Local**										
Pain	Intense	Moderate	Intense	Mild	Intense	Mild	Intense	Intense	8	
Indurated edema	Yes	Yes	Yes	Yes	Yes	Yes	Yes	Yes	8	
Livedoid plaque	Yes	Yes	Yes	Yes	Yes	Yes	Yes	Yes	8	
Size (cm/d)*	NM	5	4	NM	6	5	15	4	6	5 (4-6)
Erythema	Yes	Yes	Yes	Yes	No	Yes	No	Yes	6	
Vesicles/blisters	No	No	Yes	Yes	Yes	No	Yes	Yes	5	
Skin ulceration (Days post-bite)	12	16	12	12	7	9	26	11	8	12 (11-13)
**Systemic**										
Exanthema	Trunk	Abdomen, thighs, and pelvis	Thighs	Lower members	No	Trunk and thigh	No	Generalized	6	
Others	Fever	Fever and malaise	Fever	Fever and headache	Jaundice, dark urine, and malaise	NR	Shivering and tremors	Fever, paresthesia (hands and feet), and generalized itching	7	
**Antivenom**										
Hours post-bite	27	46	30	49	81	124	No AV used	25	7	46 (29-65)
Number of vials	5	4	5	5	5	5	-	5	7	5 (5-5)
Oral prednisone	No	No	No	No	Yes	No	No	Yes	2	
**Topical**										
Papain	No	Yes	No	Yes	Yes	Yes	Yes	Yes	6	
EFAL	No	Yes	No	Yes	No	Yes	Yes	No	4	
Mechanical debridement	No	Yes	No	Yes	Yes	No	Yes	No	4	
Healing time (Days post-bite)	34	91	ACWH (20)	53	74	ACWH (60)	98	61		68 (55-87)

ACWH: almost complete wound healing; AV: anti-arachnidic antivenom; EFAL: essential fatty acid lotion; IQR: interquartile range; NM: not measured; NR: not reported. *Largest dimension of the ischemic area (livedoid plaque) upon admission, excluding the surrounding indurated edema.

### Antivenom therapy and other treatments

Seven patients were treated with anti-arachnidic AV (median, 46 h post-bite; IQR: 29-65 h). Topical treatment involved sequential applications of papain (10% and 3%) and mechanical debridement, followed by the application of an oily lotion of essential fatty acids. Two patients were treated with oral prednisone for 5 days, one of whom had CHL (case 5) and the other, with CNL, had uncontrolled itching with no response to histamine H_1_ antagonists (case 8). Complete lesion healing was observed in six patients (Table 2) and occurred 34-98 days post-bite (median = 68 days; IQR: 55-87 days). None of the patients required reconstructive plastic surgery. 

### Photodocumentation of lesion evolution

Supplementary Material Figures 1,2,3,4,5,6,7,8 provide photographic documentation of the lesion evolution in the eight cases of this series, from the initial admission to the ED onwards. These images illustrate the slow wound healing and skin rash observed in loxoscelism, including the “typical” initial local lesion characterized by pale, ischemic, violaceous areas (livedoid plaque; Supplementary Material Figure 2, D2 and D3), a livedoid plaque and local spreading edema (Supplementary Material Figure 1, D2), a generalized scarlatiniform rash with blanching in response to digital pressure (Supplementary Material Figures 1 and 8, D2), a livedoid plaque with serohemorrhagic vesicles surrounded by a large erythematous plaque evolving with serohemorrhagic blisters (Supplementary Material Figure 4, D5-D11), a large ischemic violaceous area surrounded by a pale halo (Supplementary Material Figure 7, D9-D15), and an ischemic lesion with confluent ulcerations (Supplementary Material Figure 5, D10). Additionally, we documented the progression of the ischemic lesion to ulceration with well-defined borders (Supplementary Material Figure 6, D9-D45), the presence of granular tissue during the wound healing of a large lesion (Supplementary Material Figure 7, D57-D64), and the lesion appearance at various times (2 months to 8 years) after local lesion healing in cases 2 and 5-8 (Supplementary Material Figure 9). Hypochromic scars were observed in cases 2, 5, and 6, while case 7 showed a hypertrophic scar; tissue retraction occurred in case 8. 

## DISCUSSION

Although 53% of the 76 cases of loxoscelism admitted to our ED during the study period developed dermonecrosis, complete photodocumentation of the evolution of dermonecrosis until complete or almost complete wound healing was available for only eight patients, who were included in this study. The frequency of dermonecrosis in the patients admitted to our ED with loxoscelism was similar to previous reports from three large case series’ in the Brazilian states of Santa Catarina (1985-1996, n = 267, within the geographic range of *L. laeta*)^11^ and São Paulo (1985-1996, n = 359, and 2004-2006, n = 81; both within the geographic range of *L. gaucho*), which reported dermonecrosis in 53-58% of cases^12^ ^,^ ^13^. In contrast, the frequency of massive hemolysis was higher in Santa Catarina (13%) than in São Paulo (3-4%), indicating more severe envenomation by *L. laeta* ^11^ ^,^ ^12^. However, Málaque et al.^13^ suggested that the frequency of hemolysis in loxoscelism in geographic regions where *L. gaucho* predominates may be higher than what has been previously reported, when this manifestation is analyzed based on the increased serum levels of indirect bilirubin and reticulocyte counts; based on these criteria, the authors reported a frequency of hemolysis of up to 31%. 

As mentioned in the Introduction, the diagnosis of loxoscelism is rarely based on identification of the offending spider, as reported here. Based on the NOT RECLUSE mnemonic device^8^, all patients in the present case series fulfilled the clinical criteria for the diagnosis of probable/presumptive loxoscelism^5^. The time required for healing (within 3 months post-bite; median = 68 days) was similar to that of patients who developed more intense dermonecrosis, that is, skin necrosis >1 cm^2^ (grade 3, mean = 74 days)^6^. 

Sphingomyelinase D is the most important toxin involved in dermonecrosis and intravascular hemolysis^14^ ^,^ ^15^. This toxin activates the complement cascade, polymorphonuclear cells and platelets, in addition to inducing the expression of metalloproteinases in rabbits and human keratinocytes; metalloproteinases contribute to dermonecrosis, whereas lesion expansion or spreading is attributed to the action of hyaluronidase. Sphingomyelinase D-induced metalloproteinases cleave red blood cell surface glycophorins, leading to hemolysis and the release of hemoglobin into the general circulation^14^ ^,^ ^15^. 

Biopsies of necrotic tissue after *Loxosceles* spp. bites in humans have revealed the presence of indurated edema and an inflammatory infiltrate composed predominantly of polymorphonuclear leukocytes, as well as local thrombosis, hemorrhage, and liquefactive necrosis of the dermis and epidermis, similar to that described in pyoderma gangrenosum^4^. Similar lesions have been reproduced in animal models, such as rabbits. However, the temporal evolution of histopathological alterations in rabbits indicates faster development and spreading of cutaneous necrosis than that in humans, with cutaneous necrosis being detected at the site of venom inoculation within 24 h post-venom injection^4^; in humans, ulceration usually starts 7-14 days post-envenomation^2^ ^,^ ^3^ ^,^ ^8^. In the case series examined here, the earliest skin ulceration was detected 7 days post-bite (median, 12 days). 

Based on the current guidelines for the diagnosis and clinical treatment of loxoscelism in Brazil^10^, seven patients were treated with anti-arachnidic AV, with the earliest treated patient (case 8) receiving AV 25 h post-bite. No AV was administered in case 7 since the patient only sought the ED 9 days after the bite. Overall, the time between the bite and AV administration was very similar to the median of 48 h reported by Málaque et al.^13^

Anti-arachnidic AV has been used to treat loxoscelism in Brazil since the 1960s and shows good neutralizing potency against the dermonecrosis and lethality of various *Loxosceles* spp. venoms in rabbits^16^. However, doubts remain regarding its efficacy in neutralizing the local and systemic effects of loxoscelism in humans, what the most appropriate time for post-envenomation AV administration is, and the length of administration. Of relevance in this regard is the length of time that venom persists at the site of inoculation. Based on enzyme-linked immunosorbent assay (ELISA), *Loxosceles* spp. venoms have been shown to remain at the bite site for a considerable time after inoculation. Cardoso et al.^17^ reported two cases, one involving a 72-year-old woman and another of a 20-month-old girl, in which venom was detectable in necrotic skin lesions 5 and 29 days after the bite, respectively. In addition, an experimental study in rabbits demonstrated that *L. reclusa* venom and sphingomyelinase D were detectable at the site of injection 14-21 days after injection^18^. 

Experimental studies in rabbits have shown that when a specific anti-*Loxosceles* AV produced by the Centro de Produção e Pesquisa de Imunobiológicos do Paraná (equine origin, Fab´_2_, 1 mL neutralizes 15 minimum necrotic doses of the venom of *L. gaucho*, *L. laeta*, and *L. intermedia* in rabbits) and available for use in southern Brazil is administered intravenously 6 h, 12 h, 24 h, and 48 h post-venom, the extent of dermonecrosis induced by two minimum necrotic doses of *L. intermedia* venom (equivalent to 16 µg of venom) is significantly reduced. The greatest effect was observed in rabbits injected with AV 6 h after envenomation (90% reduction in the size of the dermonecrotic lesion when compared to the controls)^19^. 

Although controversial, the use of corticosteroids either orally, such as prednisone (40-60 mg/day/5-7 days) or parenterally (intravenous hydrocortisone or prednisolone), has been recommended for the treatment of CHL in some countries, for example, Brazil^10^, Chile^20^, and USA^5^. In addition, despite the lack of clear clinical evidence of a beneficial effect, a 5- to 7-day course of oral prednisone has been recommended for the treatment of CNL in Brazil since 2014 for patients who develop a livedoid plaque^10^. In contrast, a multivariate analysis of the clinical outcome (diameter of the necrotic area and time to heal) in a prospective series of 189 patients with loxoscelism in Oklahoma (USA, 1995-2000) revealed no significant difference between patients treated systemically with corticosteroids (n = 76) compared with non-treated patients (n = 113)^21^. However, the type of corticosteroids used and the dosage scheme were not clearly indicated in this report, nor was there a clear statement of the time after the bite when the treatment was initiated. The foregoing discussion indicates that there is clearly a need for robust clinical trials to formally assess the efficacy of AV and corticosteroids in the treatment of loxoscelism.

The topical treatments used in this study were based on the general recommendations of the university hospital´s wound treatment group, with the general aim of preventing secondary infections, stimulating angiogenesis, and accelerating wound healing. However, because of inherent limitations in the study design, it was not possible to rigorously assess whether topical treatment influenced the process of wound healing. A potentially useful novel approach for treating venom-induced local effects involves drug repurposing, specifically the topical application of tetracycline in the case of loxoscelism^15^ ^,^ ^22^. Experimental studies in rabbits have shown that the topical application of a cream containing lanolin and 5% tetracycline for up to 6 h after the injection of *L. intermedia* venom or recombinant sphingomyelinase D significantly reduced the local production of matrix metalloproteinases MPP-2 and MPP-9 and the progression of dermonecrosis^22^. These findings suggest that tetracycline could be a potentially useful drug for treating cutaneous loxoscelism, but this remains to be assessed in formal clinical trials in humans. 

The present study has several limitations, including the type of study (retrospective cohort), the small number of patients examined that reflected the strict inclusion criterion of complete photodocumentation of the case until healing or almost complete healing, and possible bias in patient selection because of this criterion. Despite these limitations, this case series provides a useful record of complete sequential photographic documentation of loxoscelism from initial admission until complete wound healing. The results indicate considerable variation in the process of wound healing, with complete epithelialization requiring up to 3 months post-bite. Despite the use of AV and rigorous treatment and care of the local lesions, all patients showed permanent local scarring of varying intensity that involved hypochromic and hypertrophic scars.
