# Supplementary material for: Temporal evolution of dermonecrosis in loxoscelism assessed by photodocumentation
Source: Rev Soc Bras Med Trop. 2022 Feb 25;55:e0502-2021. doi: 10.1590/0037-8682-0502-2021 (PMC8909434; doi:10.1590/0037-8682-0502-2021)
Supplement: Supplementary file 1 [file 1678-9849-rsbmt-55-e0502-2021-supp1.pdf]

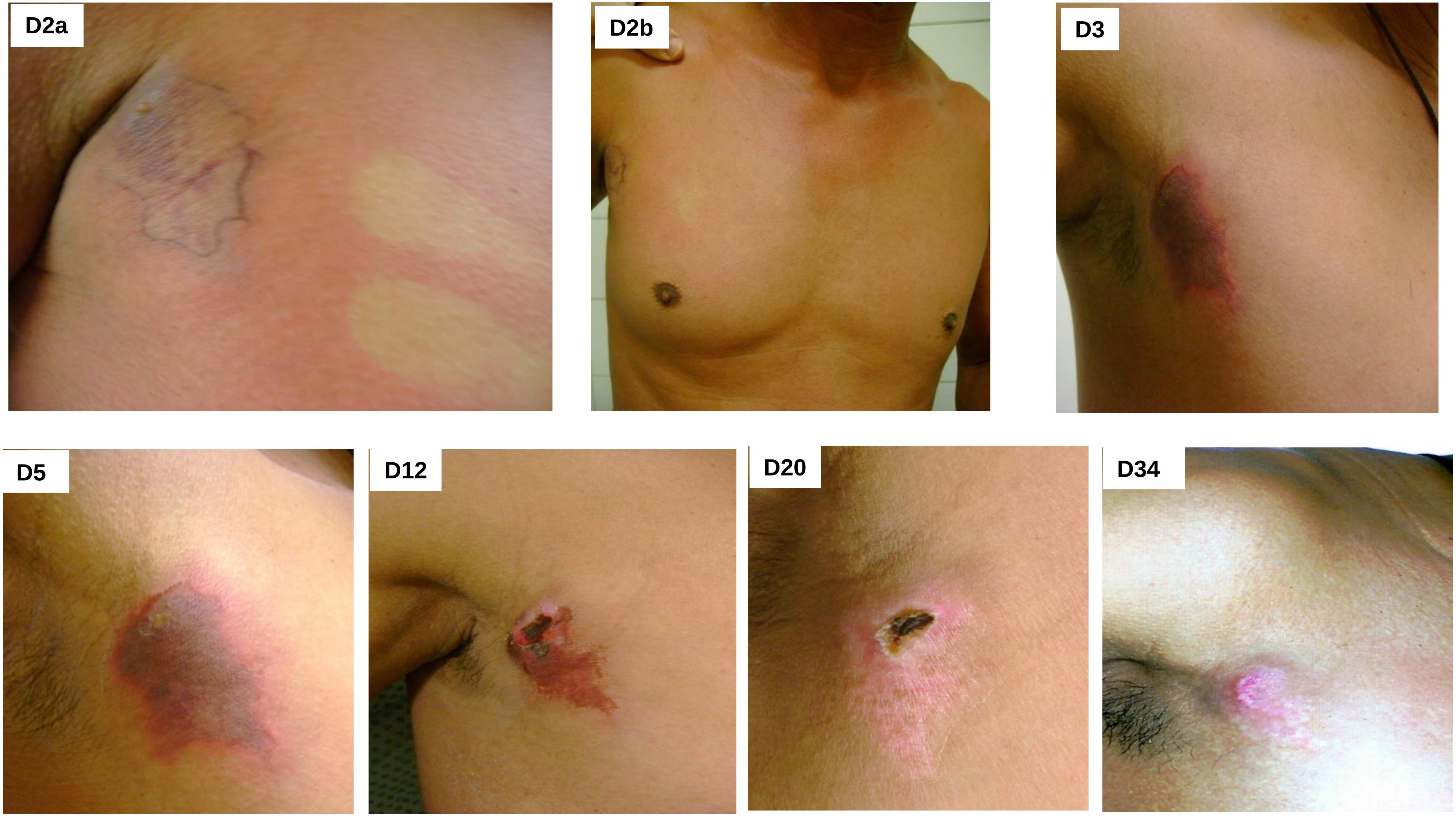

FIGURE 1. Case 1: Day 2 post-bite (D2). D2a, an irregular area interspersed with pale, violaceous areas overlying a region of indurated edema in the right periaxillary region (livedoid plaque), and scarlatiniform rash that blanched in response to digital pressure (note the two digital impressions). D2b, spreading of the local edema into the right pectoral region. D3–D20, progression of the ischemic lesion with ulceration (necrosis) from D12 onwards. D34, substitution of necrotic tissue with epithelialized tissue.
