# Supplementary material for: Temporal evolution of dermonecrosis in loxoscelism assessed by photodocumentation
Source: Rev Soc Bras Med Trop. 2022 Feb 25;55:e0502-2021. doi: 10.1590/0037-8682-0502-2021 (PMC8909434; doi:10.1590/0037-8682-0502-2021)
Supplement: Supplementary file 2 [file 1678-9849-rsbmt-55-e0502-2021-supp2.pdf]

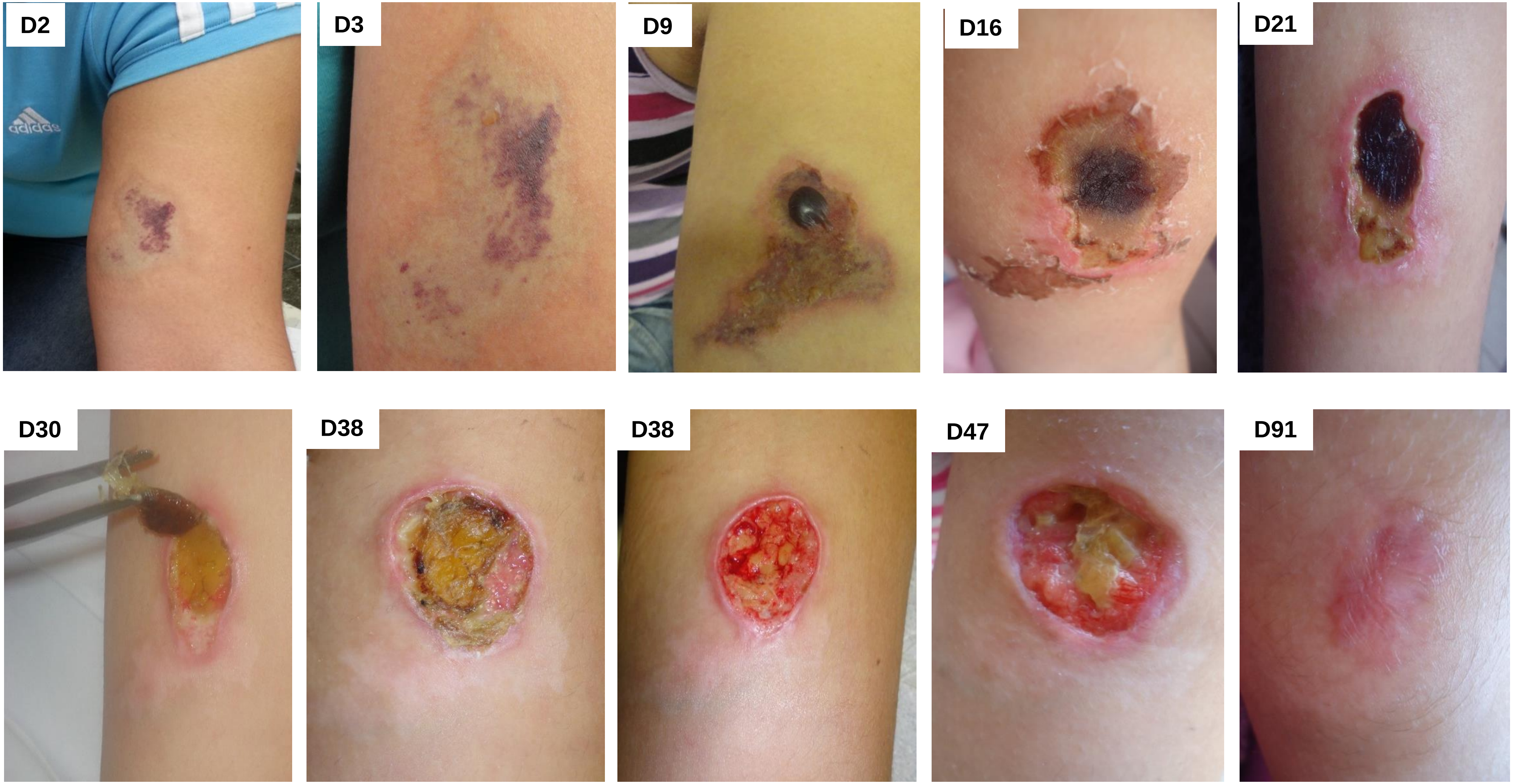


**FIGURE 2.** Case 2: Day 2 post-bite (D2), local lesion with pale, ischemic, violaceous areas overlying indurated edema (livedoid plaque) on the left arm. D3, vesicles in the livedoid plaque. D9, transformation of vesicle into a hemorrhagic blister. D16–D21, evolution of the ischemic lesion with ulceration (necrosis) and presence of dead tissue in the wound, with well-defined borders. D30, mechanical debridement. D38–D47, cicatrization with the appearance of granular tissue. D91, hypertrophic scar.
