# Supplementary material for: Temporal evolution of dermonecrosis in loxoscelism assessed by photodocumentation
Source: Rev Soc Bras Med Trop. 2022 Feb 25;55:e0502-2021. doi: 10.1590/0037-8682-0502-2021 (PMC8909434; doi:10.1590/0037-8682-0502-2021)
Supplement: Supplementary file 3 [file 1678-9849-rsbmt-55-e0502-2021-supp3.pdf]

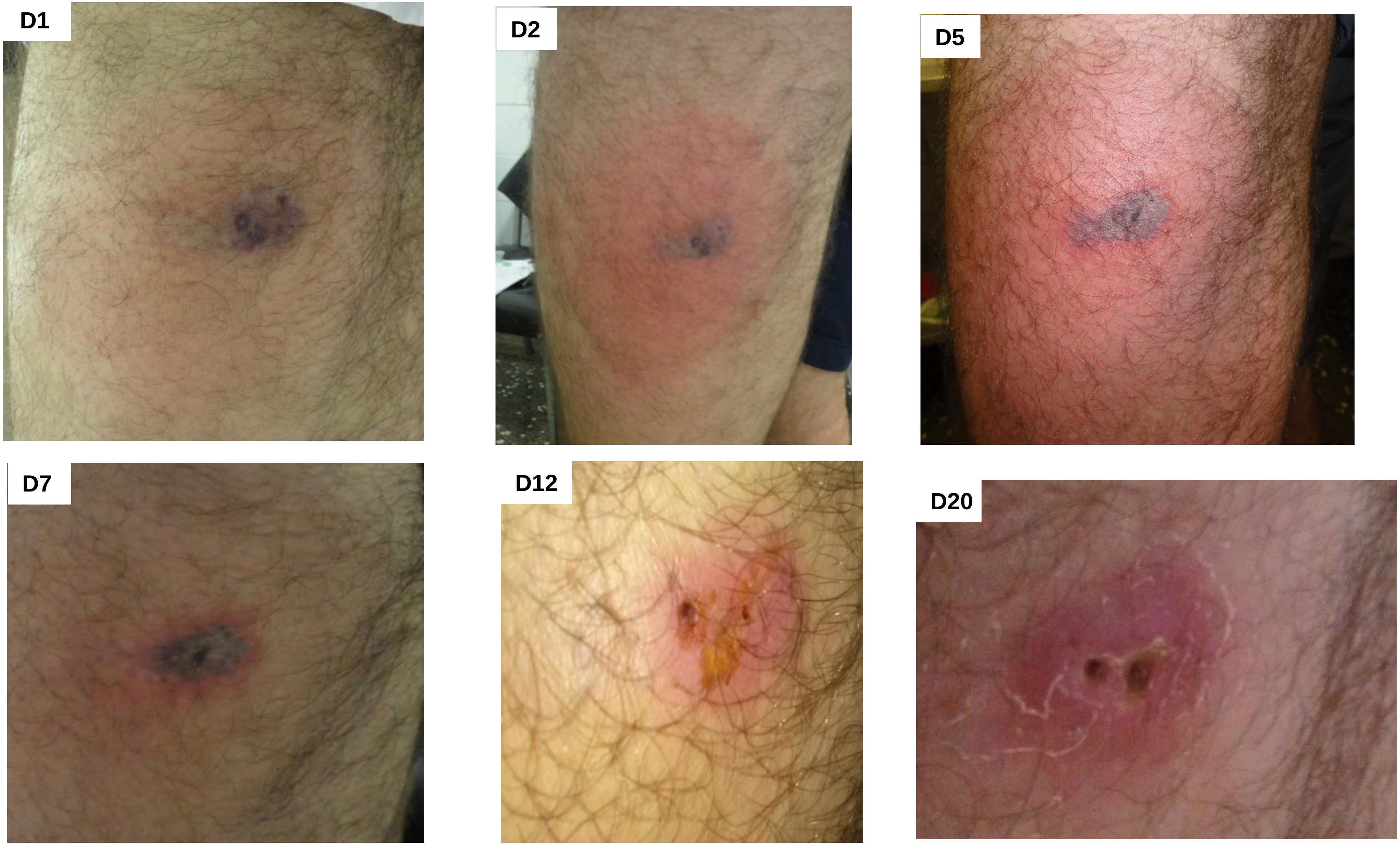

FIGURE 3. Case 3: Day 1 post-bite (D1), erythema and edema with pale, ischemic, violaceous areas (livedoid plaque) on the upper third of the right thigh. D2–D12, increase in erythema, and progression of the ischemic lesion with the occurrence of superficial ulceration. D20, healing with the substitution of dead tissue by epithelial tissue by D20.
