# Supplementary material for: Temporal evolution of dermonecrosis in loxoscelism assessed by photodocumentation
Source: Rev Soc Bras Med Trop. 2022 Feb 25;55:e0502-2021. doi: 10.1590/0037-8682-0502-2021 (PMC8909434; doi:10.1590/0037-8682-0502-2021)
Supplement: Supplementary file 4 [file 1678-9849-rsbmt-55-e0502-2021-supp4.pdf]

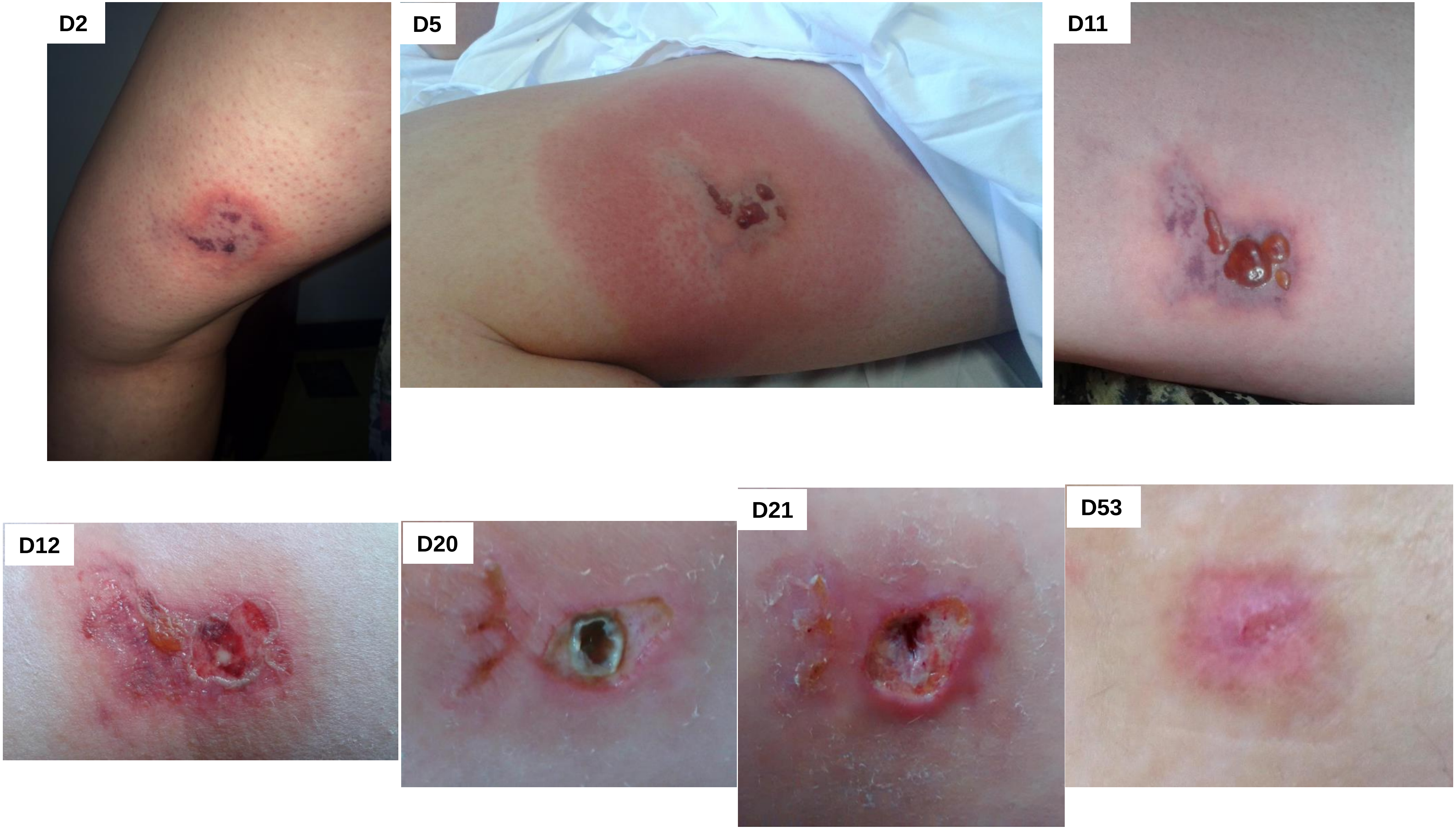

FIGURE 4. Case 4: Day 2 post-bite (D2), erythema, edema, pale, ischemic, violaceous areas (livedoid plaque) and hemorrhagic vesicles in the medial third of the right thigh. D5–D11, increase in the ischemic area and in the erythematous plaque, with the presence of serohemorrhagic vesicles/blisters. D12–D21, ulceration. D53, epithelialized tissue.
