# Supplementary material for: Temporal evolution of dermonecrosis in loxoscelism assessed by photodocumentation
Source: Rev Soc Bras Med Trop. 2022 Feb 25;55:e0502-2021. doi: 10.1590/0037-8682-0502-2021 (PMC8909434; doi:10.1590/0037-8682-0502-2021)
Supplement: Supplementary file 5 [file 1678-9849-rsbmt-55-e0502-2021-supp5.pdf]

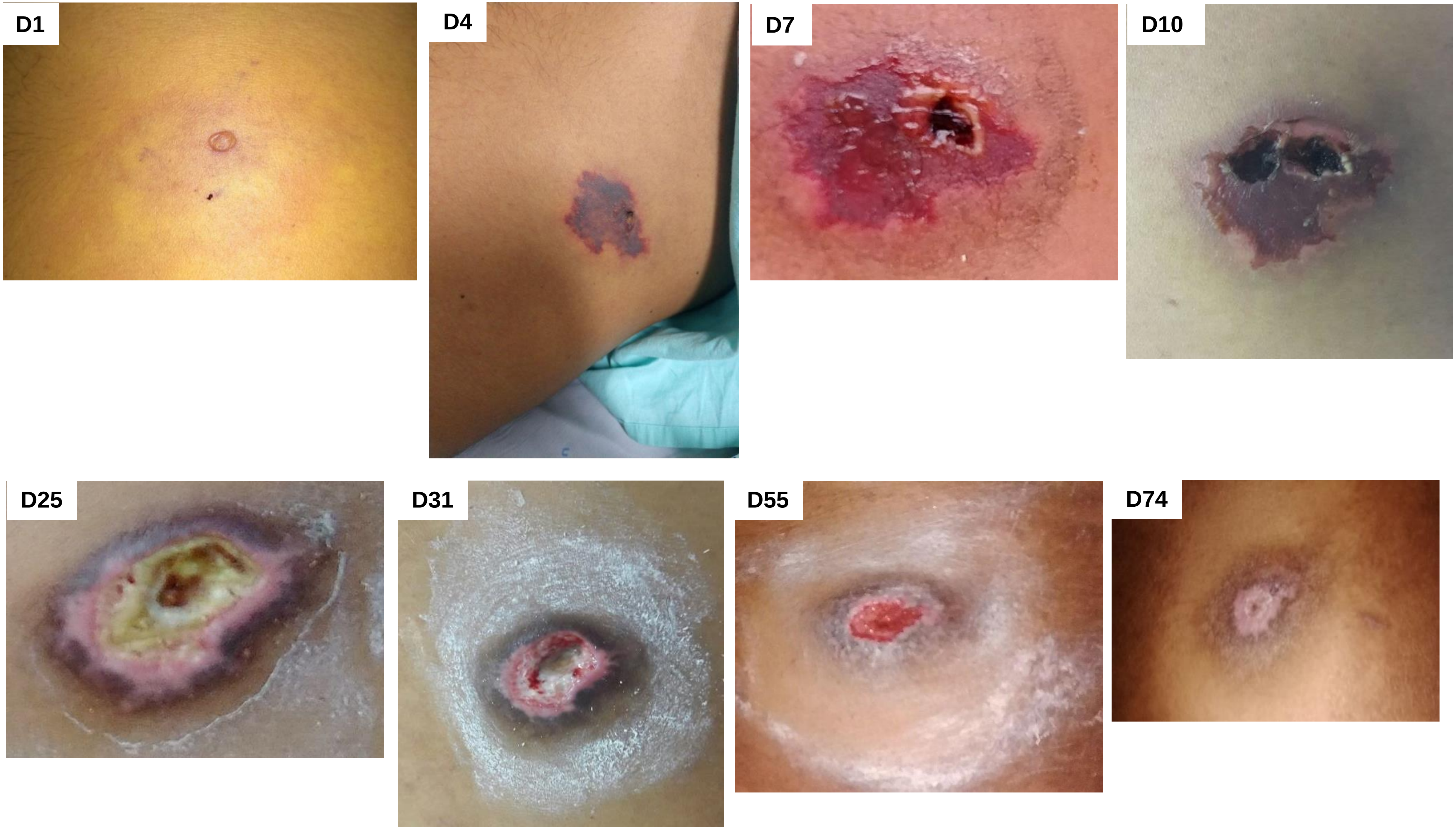

FIGURE 5. Case 5: Day 1 post-bite (D1), edema, pale areas, and a vesicle in the central region. D4, increase in lesion size, with a pale halo surrounding a livedoid plaque. Note the burst hemorrhagic vesicle at the periphery of the lesion. D7–D10; progression of the ischemic lesion with ulceration (necrosis). D25–D31, sloughing in the lesion bed. D55, lesion with granular tissue. D74, epithelialized lesion.
