# Supplementary material for: Temporal evolution of dermonecrosis in loxoscelism assessed by photodocumentation
Source: Rev Soc Bras Med Trop. 2022 Feb 25;55:e0502-2021. doi: 10.1590/0037-8682-0502-2021 (PMC8909434; doi:10.1590/0037-8682-0502-2021)
Supplement: Supplementary file 6 [file 1678-9849-rsbmt-55-e0502-2021-supp6.pdf]

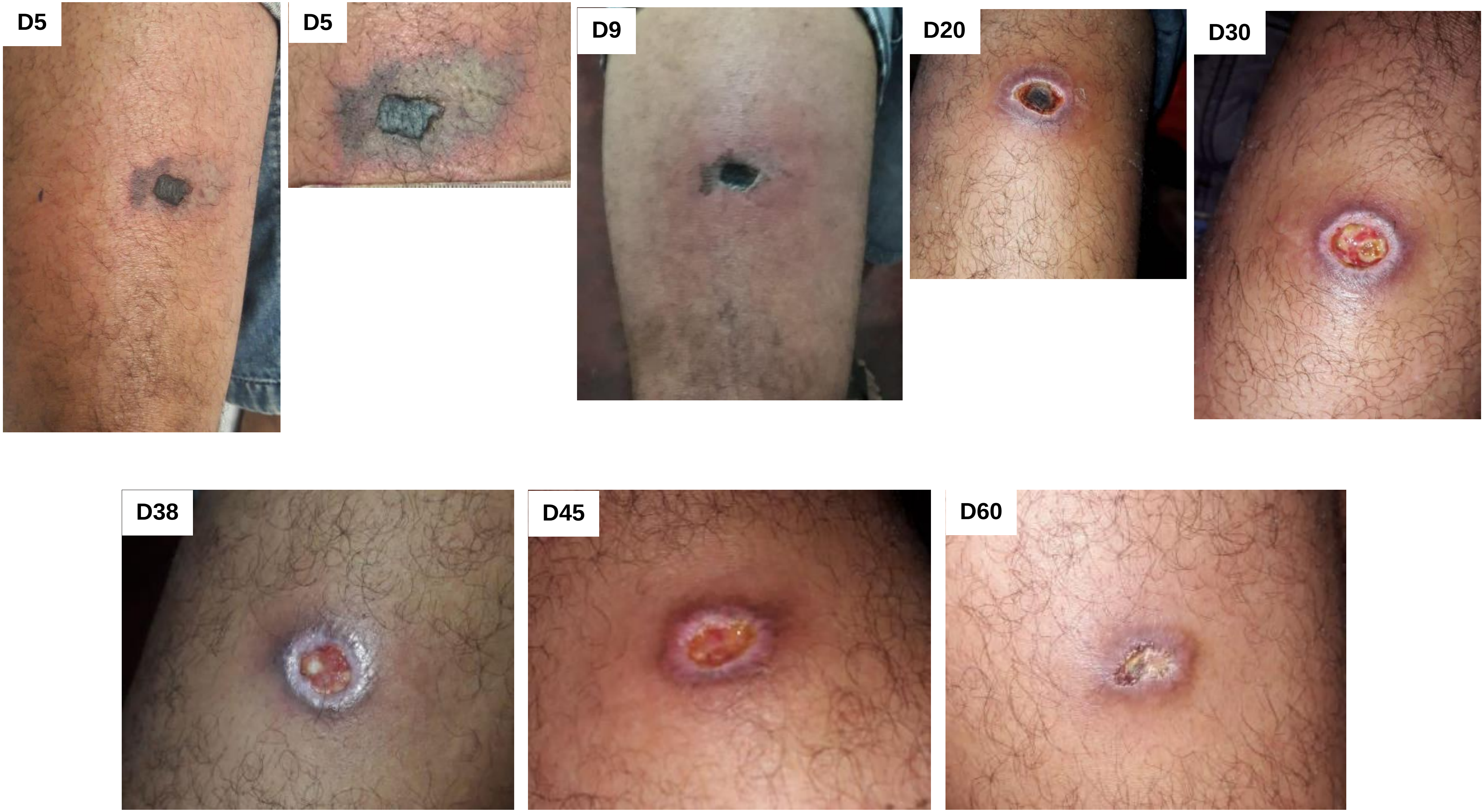

FIGURE 6. Case 6: Day 5 post-bite (D5), pale, ecchymotic and ischemic violaceous areas overlying a region of indurated edema (livedoid plaque), with a hemorrhagic blister in the middle. D9, progression of the ischemic lesion, with the onset of ulceration (necrosis). D20–D45, progression of cicatrization showing a well-defined border and dead tissue within the lesion. D60, partially epithelialized lesion.
