# Supplementary material for: Temporal evolution of dermonecrosis in loxoscelism assessed by photodocumentation
Source: Rev Soc Bras Med Trop. 2022 Feb 25;55:e0502-2021. doi: 10.1590/0037-8682-0502-2021 (PMC8909434; doi:10.1590/0037-8682-0502-2021)
Supplement: Supplementary file 7 [file 1678-9849-rsbmt-55-e0502-2021-supp7.pdf]

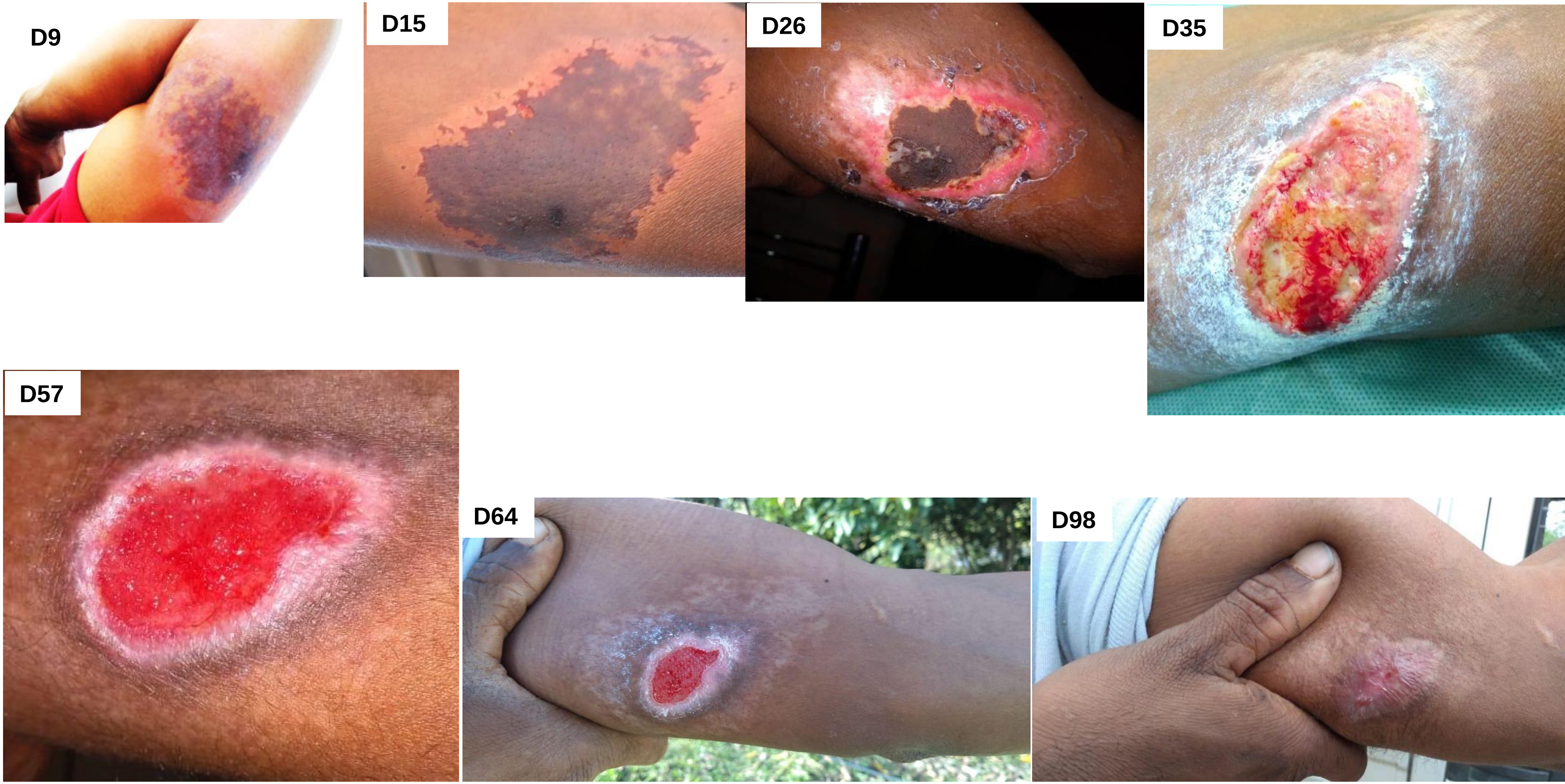

FIGURE 7. Case 7: Day 9 to day 15 post-bite (D9–D15), a large ischemic violaceous area surrounded by a pale halo on the left arm, with hemorrhagic vesicles at the periphery. D26, progression of the ischemic lesion with necrosis in the central region. D35, sloughing in the lesion bed. D57–D64, lesion with granular tissue. D98, hypertrophic scar.
