# Supplementary material for: Temporal evolution of dermonecrosis in loxoscelism assessed by photodocumentation
Source: Rev Soc Bras Med Trop. 2022 Feb 25;55:e0502-2021. doi: 10.1590/0037-8682-0502-2021 (PMC8909434; doi:10.1590/0037-8682-0502-2021)
Supplement: Supplementary file 8 [file 1678-9849-rsbmt-55-e0502-2021-supp8.pdf]

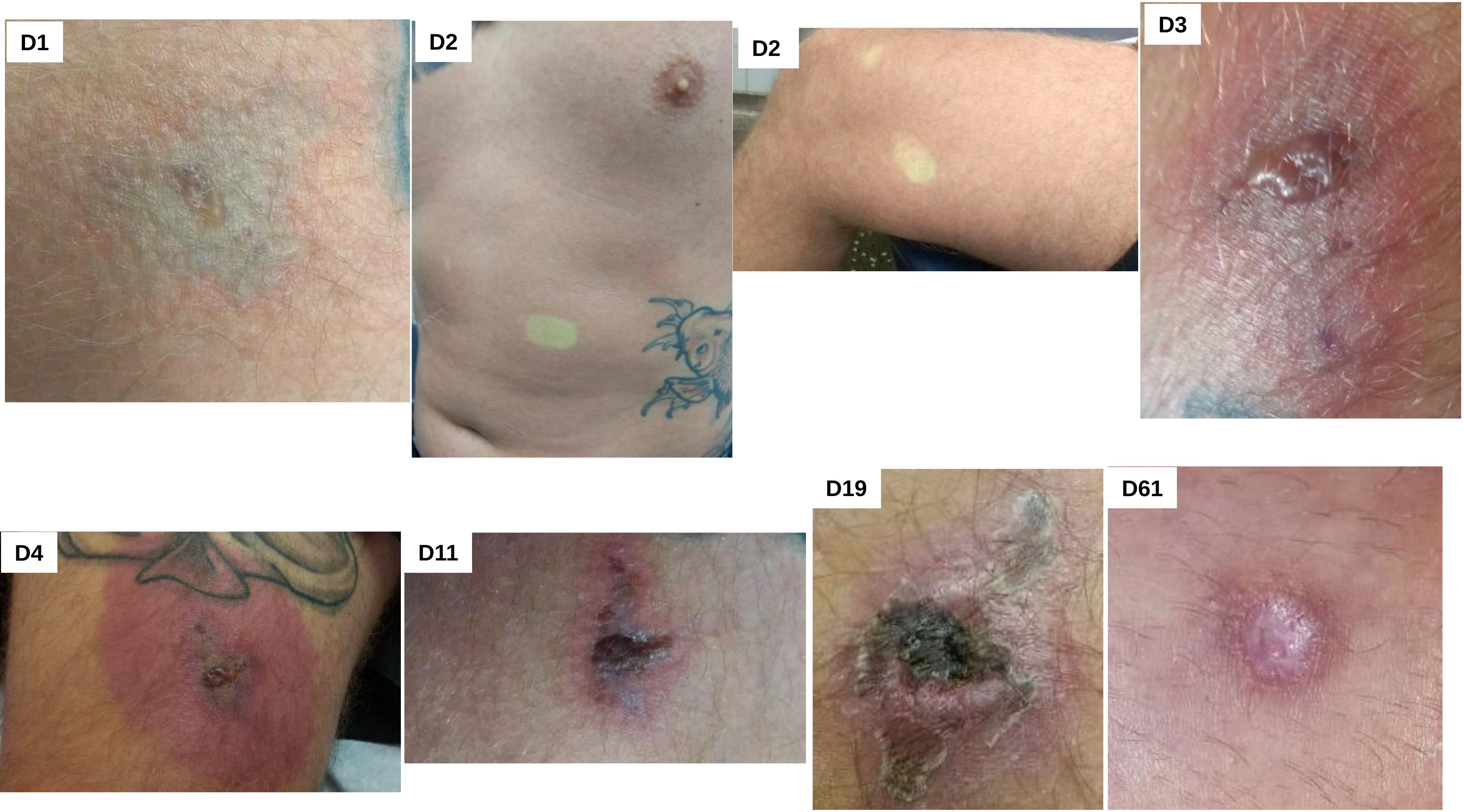

FIGURE 8. Case 8: Day 1 post-bite (D1), local lesion with edema, erythema, and violaceous areas on the upper third of the left thigh, with vesicles in the central region of the lesion; generalized scarlatiniform rash with blanching in response to digital pressure. D3, progression of the ischemic lesion with an increase in vesicle size. D4, increase in the erythematous region and ischemic area. D11–D19, definition and evolution of the necrotic area. D61, epithelialized lesion.
