# Supplementary material for: Temporal evolution of dermonecrosis in loxoscelism assessed by photodocumentation
Source: Rev Soc Bras Med Trop. 2022 Feb 25;55:e0502-2021. doi: 10.1590/0037-8682-0502-2021 (PMC8909434; doi:10.1590/0037-8682-0502-2021)
Supplement: Supplementary file 9 [file 1678-9849-rsbmt-55-e0502-2021-supp9.pdf]

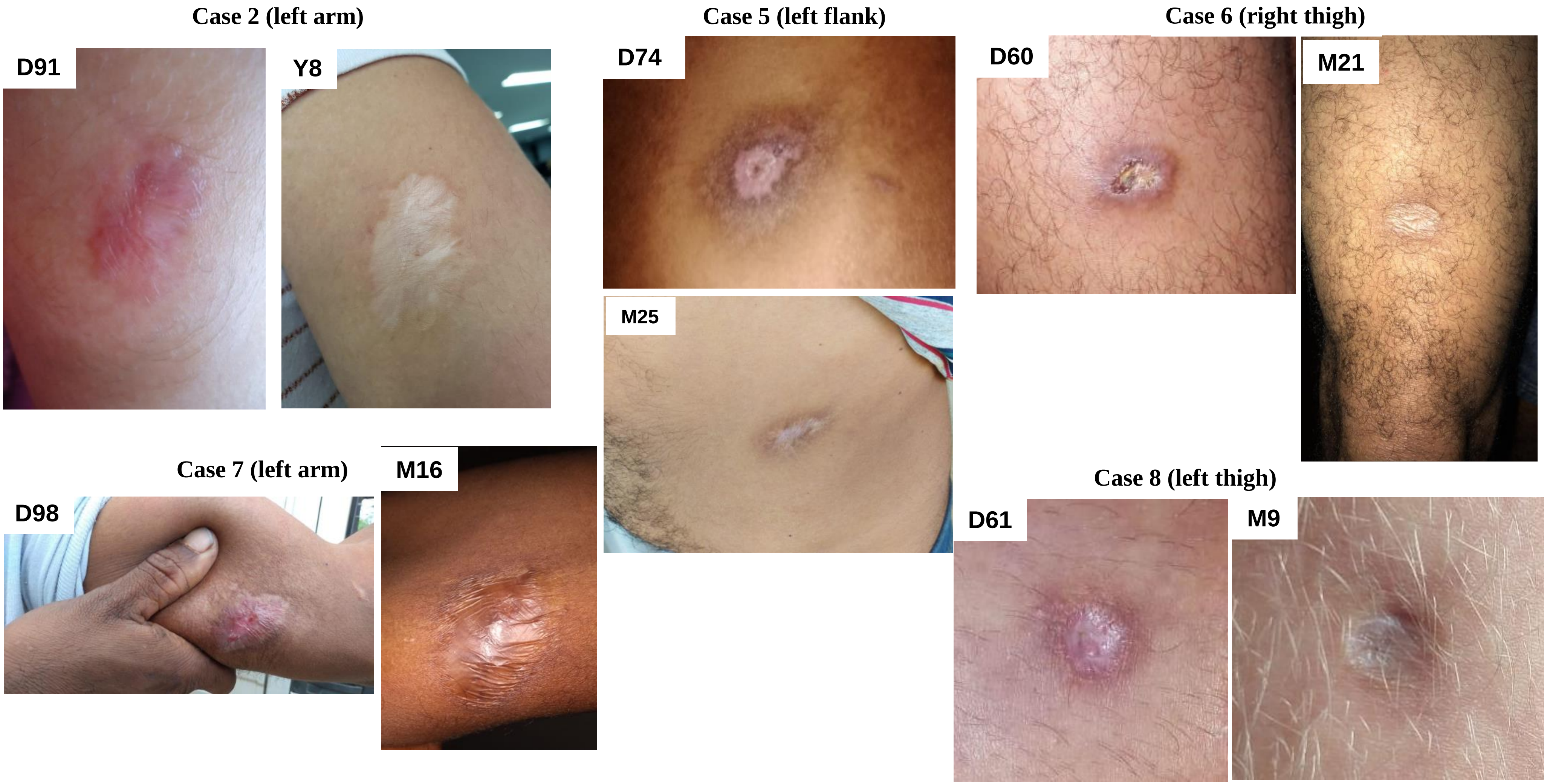

FIGURE 9. Final aspect of the lesions months (M) and years (Y) after the bite compared with the last evaluation indicated in Figures 2 and 5–8 for cases 2 and 5–8, respectively. Note that patients 2, 5 and 6 evolved with hypochromic scars, patient 7 had a hypertrophic scar and patient 8 showed tissue retraction.
